# Preface of the Special Issue: “Recent CMV Research”

**DOI:** 10.3390/v6010336

**Published:** 2014-01-22

**Authors:** Kayla Dufrene, Roberta L. DeBiasi, Anamaris M. Colberg-Poley

**Affiliations:** 1 Gallaudet University, 800 Florida Avenue NE, Washington, DC 20002, USA; E-Mail: kayla.dufrene@gallaudet.edu; 2 Departments of Pediatrics, Children’s National Medical Center, 111 Michigan Avenue, NW Washington, DC 20010, USA; E-Mail: rdebiasi@childrensnational.org; 3 Microbiology, Immunology and Tropical Medicine, George Washington University, Washington, DC 20037, USA; 4 Integrative Systems Biology, Research Center for Genetic Medicine, Children’s National Medical Center, 111 Michigan Avenue, NW Washington, DC 20010, USA; 5 Biochemistry and Molecular Medicine, School of Medicine and Health Sciences, George Washington University, Washington, DC 20037, USA

## 1. Foreword

**Figure 1:**
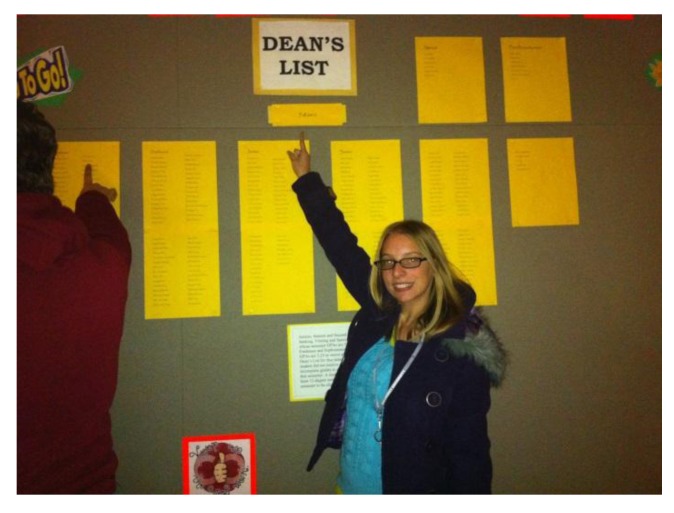
Kayla finding out that she made the Dean’s List at Gallaudet University.

This ***Viruses*** Special Issue on Recent Cytomegalovirus (CMV) Research is dedicated to the patients who have suffered CMV infection and to their parents, families and caregivers. We are including as a Preface to this issue the insights of a young college student, Kayla Dufrene, who suffered congenital CMV infection and contacted me and Dr. Roberta DeBiasi, to interview us to learn more about CMV. As I was just returning to the DC area from the 4th Congenital CMV Conference in San Francisco, I was particularly receptive to her request. When we met Kayla, we were both impressed with her personal strength and ability to cope with her disabilities and needed medical treatments. Despite it all, Kayla has an exceptionally positive outlook on life, feeling even lucky. She has not only coped, but has transcended her difficulties. I am proud to say that she was on the Dean’s List (Figure 1) at Gallaudet University. Ultimately, her hope lies in our fields’ efforts to develop a vaccine to prevent CMV disease in other children. 

**Figure 2:**
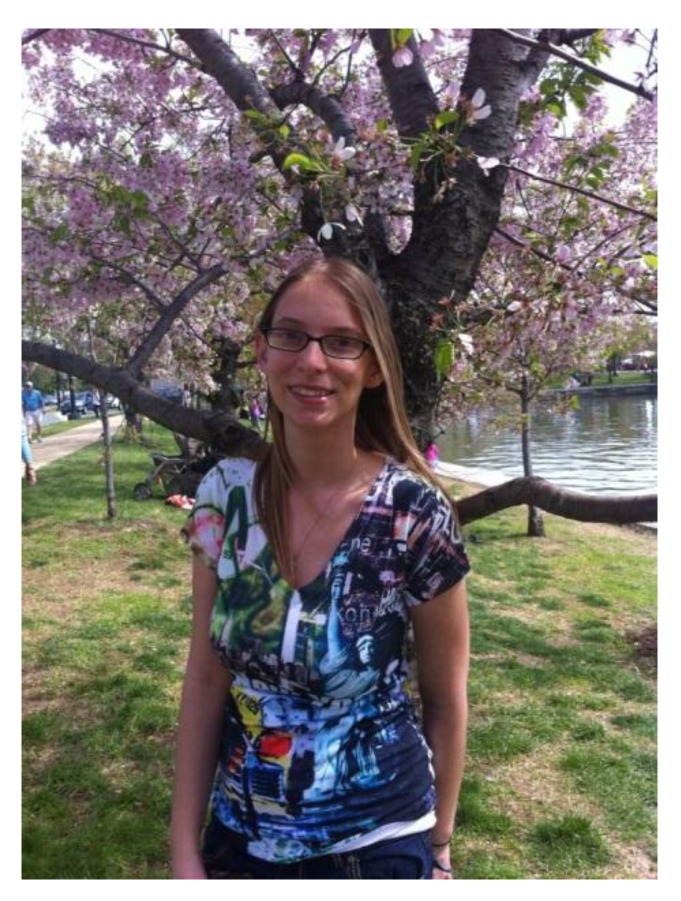
Kayla enjoying the cherry blossoms.

Her autobiography (in her own words) is our Preface. For those of us who work on the virus and anyone interested in the consequences of CMV disease, it is a touching and inspiring read (Figure 2). 

Anamaris Colberg-Poley, Guest Editor, Viruses, Special Issue, Recent CMV Research and Roberta L. DeBiasi, Professor of Pediatrics, GWU, Acting Chief, Division of Pediatric Infectious Diseases, Children’s National Medical Center.

## 2. Preface: Cytomegalovirus — Patient Monograph — Kayla Dufrene

Prior to writing a research paper for a college assignment, I never felt the need to learn more about Cytomegalovirus (CMV). I didn’t know a lot about CMV, I just grew up hearing my Mom tell doctors that it’s what I was born with. It’s the reason why I have hearing loss, bad eyesight, and muscle problems in my legs, and also the cyst in my brain. It’s also why I have had to endure two eye surgeries and surgery on both my hips. When I was born, I was very sick. Besides having CMV I had an enlarged liver (when the liver swells beyond its normal size) and yellow jaundice (yellowing of the skin). I was very tiny and had to stay in the hospital for a month. The doctors told my birth parents I would either die or not have a good quality of life. The decision was then made to put me up for adoption.

It turns out I definitely DID want to know more about CMV. What are the symptoms? Is there a cure? Is this a genetic disease? Is there genetic testing? How does it affect my body? How does someone get CMV? Is it an STD? How is it diagnosed? Can I get it later in life? Is there a vaccine for infants? Is there a test for CMV? Is it contagious? Who can get CMV? How many people are diagnosed with cytomegalovirus? How has it affected me? Do I still have it in my system? How does my case compare to others? How will it affect me later in life? Could it affect my sex partner? Could I really have died? Did it affect my birth mom? Is it a genetic disease and something I have to worry about in the future?

After reading texts and online references on Gallaudet’s library webpage, I had a foundation for my search and I was ready to start building from there. I developed a desire for more knowledge and answers to a more of my questions. I learned that CMV is a more popular subject than I thought. When I read about the impact it can have on the family, it made me understand a little more why my birth parents put me up for adoption. They couldn’t have known the extent that I would be affected yet here I am, 19 years later, a student at Gallaudet University. I was very excited to continue my search and learn more about the disease that has made me the person who I am today. 

My reading taught me that development of a CMV vaccine was a national top priority and I wanted to learn more about the efforts that doctors and researchers are going through to make a vaccine a reality. I know firsthand the effects of CMV: Knowing a vaccine could have prevented a lot of what I had to go through and could prevent newborns in the future from being affected by CMV I believe is worthwhile. CMV is very common and the effects it can have on a child and the family can be very hard. I know these from my own experiences of being picked on in school for wearing a hearing aid and being “the girl who walked a little funny”. I was excited to read about clinical trials that are testing potential vaccines for CMV. One of the articles explained a clinical trial to develop a vaccine to prevent CMV among mothers and infants. The possibility of a vaccine is very real. CMV has affected my life from the surgeries to the teasing in school and the thought that a vaccine could have prevented all of that is mind-boggling. My life could have turned out differently. 

My reading sparked my interest in interviewing someone knowledgeable about CMV research and disease. The deadline for having an interviewee was quickly approaching. I thought long and hard about where I could find someone whom I could interview. I then thought that since CMV is found in babies, maybe I should ask a pediatrician. I found out that the Children’s National Medical Center was just a few metro stops from Gallaudet and I was lucky to identify both a scientist (Dr. Colberg-Poley) and a doctor (Dr. Roberta DeBiasi) who focus their careers in this very area. They were both more than happy to meet with me. On the day of the interview, I was really nervous and wanted to make sure I had everything I needed prepared. I explained to them that I was writing a paper on a topic that had to relate to me so I chose CMV. I told them how I was born with CMV and of how it was the reason I had two eye surgeries when I was a kid, a hip surgery on both hips, and why I wear glasses and a hearing aid. They were thrilled to have me there, eager to share their knowledge about CMV, and just as excited to meet and speak with me as I was excited to meet with them. 

Dr. DeBiasi shared that in her entire career, she had never met someone as an adult who had been diagnosed with CMV as a baby. She said it was a good experience for her and an honor to meet me. I shared many of my frustrations, such as the fact that no one figured out I was deaf until third grade, and I was able to ask if I born deaf or if it was detected late. I learned that even though the virus is latent in my body (just like anyone else who is infected with CMV at any time in their life); it is not something I have to worry about when I have a sexual relationship or children. I learned that it is not a genetic illness, and not something that I am going to pass on to my children genetically. I also learned that it is hard for doctors to accept that there are diseases they can diagnose, but for which they can’t do anything about, and they want to help change that.

The one big thing that I took away was that I’m going to be okay and I do not have to stress about my future like wondering if CMV was going to affect my future children. I also realized that, when my birth mom put me up for adoption, she couldn’t have known what the future held for me. I’m glad that she’s okay. My adoptive mom did a very brave thing of adopting a baby that she knew was sick and never bat an eye and, over the last 19 years, I have not once heard her complain. Hearing Dr. Colberg-Poley and Dr. DeBiasi describe how there are even worse possible outcomes for people with CMV makes me feel extremely lucky. Something so small created a lot of problems for me growing up but it has made me a stronger person and more understanding of other people who have other problems. If it wasn’t for cytomegalovirus I wouldn’t have hearing problems and I wouldn’t be at Gallaudet University and I never would have had the chance to find out all of this information about CMV. 

Doing the interviews was the most beneficial thing to me because I have never had anyone to answer my questions. I was not sure where my search would lead me and what I would find out. I have always lived with the fear of not knowing how Cytomegalovirus would affect my future. I have always had questions like: What are the symptoms? Is there a cure? Is this a genetic disease? Do I still have it in my system? How does my case compare to others? How will it affect me later in life? Could it affect my sex partner? But I never had any answers. Now I have all the answers to all the questions I have ever asked. The search was hard and stressful but I’m glad I had the opportunity to finally learn about CMV. I have now made contact with people of whom I can ask questions that may arise later. During the interviews I learned things that I could not get through any article or any book and I got real answers. It’s amazing that something as small as a tiny virus could have such a huge impact and effect on my life. 

After my reading and interviews, I left feeling much more knowledgeable about CMV. Dr. Colberg-Poley and DeBiasi answered all of my questions and made me leave with a new-found confidence. I’m going to be okay in the future and so will my kids. CMV affected me as a baby and growing up it caused me a lot of problems, but it’s because of having Cytomegalovirus that I’ve had the experiences I’ve had and why I’m at Gallaudet University. When I started my research, I was not sure where it would lead me or what I would find. I have learned a lot about the virus and a lot about myself and just because the past was tough, it doesn’t mean the future has to be. 

